# Comparison of health-related quality of life in atopic dermatitis, hidradenitis suppurativa, pemphigus and psoriasis

**DOI:** 10.1007/s00403-024-03786-4

**Published:** 2025-01-18

**Authors:** Péter György Balázs, Krisztián Gáspár, Hunor L. Gergely, Krisztina Hajdú, Péter Holló, Kamilla Koszorú, Adrienn Katalin Poór, Miklós Sárdy, Andrea Szegedi, Béla Tamási, Norbert Wikonkál, Valentin Brodszky

**Affiliations:** 1 Institute of Social and Political Sciences, Corvinus University of Budapest, Budapest, Hungary; 2 Faculty of Medicine, University of Debrecen, Debrecen, Hungary; 3 Faculty of Medicine, Semmelweis University, Budapest, Hungary

**Keywords:** HRQoL outcome measure, Health utility measurement agreement, Atopic dermatitis, Hidradenitis suppurativa, Pemphigus, Psoriasis

## Abstract

This study aims to explore the measurement agreement between direct and indirect health utility measures in four chronic dermatological conditions (atopic dermatitis, hidradenitis suppurativa, pemphigus, psoriasis). Outpatients survey data collected between 2015 and 2021 were analysed. Health-related quality of life (HRQoL) outcome measures included time trade-off (TTO), EQ-5D-5L and Dermatology Life Quality Index (DLQI). Descriptive statistics were computed for the pooled sample and four diseases. Mean, standard deviation (SD), median, interquartile range (IQR), ceiling and floor effects were calculated for TTO, EQ-5D-5L and DLQI utilities. Bland‒Altman plots and intraclass correlation coefficients (ICC) were applied to investigate the agreement between health utility measures. Sociodemographic characteristics (age, sex, educational level, employment status) and health-related information (disease duration, outpatient care visits in the past 3 months and disease severity) impact on utilities was investigated by Tobit regressions. The sample includes N = 765 patient responses with a mean age of 41.5 (SD = 16.2), majority being males (52.7%). Total sample mean utilities were the highest according to TTO (0.83), followed by EQ-5D-5L and vDLQI (0.81 and 0.81) and lowest in mDLQI (0.77). Measurement agreement was found only between TTO and EQ-5D-5L. Skin-disease severity impacted all health state utilities, though only TTO differentiated utility values according to disease type. The discrepancies between the TTO and DLQI warn to compare DLQI-based utilities in different dermatological conditions with extreme caution.

## Introduction

Health-related quality of life (HRQoL) in chronic dermatological diseases is well studied. To different extent, skin conditions are associated with negative impact on HRQoL by causing e.g. pain, functional impairment, anxiety or social stigmatization that may lead to develop further comorbidities [1, 2]. In adult populations, chronic plaque psoriasis and atopic dermatitis are among the most common chronic dermatological conditions, while the conditions having the largest impact on HRQoL include hidradenitis suppurativa and pemphigus [3–5].

Atopic dermatitis is characterized by dry, inflamed skin lesions and intense pruritus and is very common among all population groups and ages worldwide (1.2–17.1%) [6, 7]. Hidradenitis suppurativa is a less prevalent (globally 0.7–1.2%) immune-mediated disease, causing recurrent abscesses, fistulas and scars [8]. Psoriasis, in addition to red and painful itching skin, is associated with psychological effects on self-esteem and depression and affects approximately 1–3% of the world’s population [9, 10]. Pemphigus is a rare disease of the skin and mucosa, and its global prevalence ranges between 60–95 million [11].

The measurement of HRQoL is particularly important in health economic evaluations to assess the health gain due treatments. Several health assessments require the expression of health gains (benefit) as quality-adjusted life years (QALYs) composed of quality (HRQoL) and quantity of life (years lived) [12]. The HRQoL component of the QALY is measured in terms of health state utility (HSU) applying either direct [6, 13], indirect generic [14] or indirect disease-specific [15] utility measurement methods. Health resource allocation decisions rely on QALY outcomes, therefore understanding the differences between different health state utilities is crucial. In general, direct methods often output significantly higher HSU values than indirect instruments [16, 17], but in many dermatological conditions, this evidence is contradictory [9]. Sociodemographic characteristics (age, sex, educational level or marital status) may also influence HSU [18]. Older age, being married, and strong religious beliefs are reported as factors rounding HSU estimates upwards [19, 20].

HSU measurements have two methods: The main difference between direct and indirect measurement rest in the patient’s valuation of different health states, by expressing individual HRQoL preferences through an iterative choice (directly) or by a standardized rating of pre-defined health domains (indirectly). More complex direct methods provide less cross-comparable but individual valuations about the overall health state, while simpler indirect methods generalizable rate given health domains, but without considering responding patients’ preferences [21]. Direct HSU measures mostly apply the time trade-off (TTO) method, that rely on eliciting preferences of respondents by offering a choice between a shorter full and a longer imperfect health state (decision-making theory), assuming that people can clearly choose between two alternatives (e.g.: live 10 years in psoriasis or 8 years in full health) [22, 23]. The utility value is anchored between ‘1’ expressing full health and ‘0’ meaning death, whereby positive values refer to better-than-dead (BTD) health states and negative utility values represent worse-than-dead (WTD) health states [15].

Indirect (preference-accompanied) measures such as EQ-5D-5L/3L, Health Utility Index (HUI) or Short Form 6D (SF-6D) consist of two parts: (1) a descriptive system of items and (2) a value set/mapping algorithm. The health descriptive system can be generic such as the EQ-5D or disease-specific like the Dermatology Life Quality Index (DLQI) or Skindex-16. At present, the EQ-5D is the most commonly used generic health state measure to calculate indirect HSU values, used in 29 + countries for health policy decision making [24–26]. Skin condition-specific measures use dermatology-related domains (e.g., painful skin, itching, sexual difficulties, annoyance/frustration due skin symptoms) to assess HRQoL, where the most popular tool in atopic dermatitis, hidradenitis suppurativa and psoriasis is by far the DLQI [27–29]. The item responses can be transformed into utilities, by weighting the health domains, usually using country-specific value sets (tariffs), that are based on direct utility valuations representing societal preferences of a large generic populations instead of patient’s desire. Mapping is a statistical method that uses a predictive model to link item responses to utility scores, enabling the cross-comparison of intervention effect even it was measured with different methods. The DLQI item scores similarly to EQ-5D can be converted into indirect utilities using a value set or mapping algorithms. The comparison of generic and disease-specific indirect utilities would be particularly relevant in dermatology, where only one DLQI value set coexists with multiple mapping algorithms [30–34].

Researchers may choose various direct/indirect utility measures, but the measurement tool is likely to impact the HSU estimate. Several studies have proven poor agreement between TTO and EQ-5D with wide limits of agreement, indicating that these utility measures cannot be used interchangeably [9, 35–39]. Indirect instruments tend to be similar on the level of an individual’s absolute agreement (observing whether different measurements assign the same score to the same subject). Empirical studies found strong agreement between the EQ-5D-5L and EQ-5D-3L, HUI, and SF-6D [40, 41], although some studies call for further investigations [42, 43]. There are revealed measurement inconsistencies (lack of agreement) between direct and indirect utility elicitations in dermatology [9], and in other diseases [35, 44] with limited evidence of indirect generic to indirect disease-specific methods agreement. More studies recommend using dermatology-specific instead of generic HRQoL measurements, due to sensitivity/responsiveness to change [45–47]. Our study provides a comparison of direct, and indirect generic/indirect dermatology-specific measurements.

Discrepancies in HSU scores by different methods raise questions regarding the interpretation and comparison of utility scores, affecting both aspects of economic evaluations (incremental health gain of therapies and value for money). This research aims to investigate the agreement between direct, indirect generic and disease specific (TTO-EQ5D-DLQI) utility assessment methods in four chronic dermatological conditions (atopic dermatitis, hidradenitis suppurativa, pemphigus, psoriasis). We also aimed to compare the association of sociodemographic factors and clinical characteristics with HSU.

## Methods

### Data collection

Data were pooled from four multicentre cross-sectional surveys, collecting information between 2015 and 2021 from adult patients with atopic dermatitis, hidradenitis suppurativa, pemphigus and psoriasis in Hungary. The sample consists of patients aged 18 ≤ at any disease severity stage diagnosed (according to International Classification of Diseases-10) with (i) atopic dermatitis, (ii) hidradenitis suppurativa, (iii) different types of psoriasis and (iv) pemphigus including foliaceus or vulgaris. The mode of data administration was interviewer assisted (self-completed) paper-based questionnaires, during respondents’ specialized care hospital visits. The detailed study methods were published in the previous articles of our research team [8, 11, 40, 48–57].

### Health state utility measurement

Four different HSU assessment methods were employed in each study: (1) conventional TTO (direct) [58], (2) EQ-5D-5L (indirect) [25], (3) DLQI mapped onto EQ-5D-3L (indirect: mDLQI) [30] and (4) value set-based DLQI (indirect: vDLQI) [31]. Table 1 provides a comparison of the characteristics of these measures.

**Table 1:** Typology of applied health state utility measurements

Utility measurement	Direct	Indirect		
Measurement tool*	TTO	EQ-5D-5L	vDLQI	mDLQI
Type of measure	N/a	Generic	Dermatology-specific	
Health utility valuation method	Conventional 10-year TTO	Composite TTO (EQ-VT)	Conventional 10 year TTO	Mapping of DLQI items scores into EQ-5D-3L utility
Preferences	Patient	Societal		
Country of origin	Hungary	Hungary	Hungary	United Kingdom
Theoretical range**	0–1.0	− 0.85–1.0	0.57–0.87	0.24–0.98

*TTO time trade-off, *mDLQI* mapping-based dermatology life quality index, *vDLQI* value set-based dermatology life quality index

**The theoretical range of the EQ-5D-5L values was truncated at 0 to harmonize the measurements

*(1) TTO* is the most common direct utility assessment method, where respondents swap quantity to quality of life [22, 59]. The task offers a choice living *t* years in a given imperfect health state or *t-x* years in full health. The utility is calculated from the indifference point (t-x/t = u), where the respondent has equal preference towards the two health states. For instance, if the respondent has to choose between living 10 years in plaque psoriasis or living 10-x years in full health and the traded years are x = 2, the conventional TTO utility is given as *u* = *(10–2)/10* = *0.8*. TTO method in two studies (atopic dermatitis, psoriasis [55, 56]) was conventional, in case of hidradenitis suppurativa and pemphigus studies [8, 11], composite TTO was used.

*(2) The EQ-5D-5L* generic HRQoL measure, consisting of five dimensions: mobility, self-care, usual activities, pain/discomfort, and anxiety/depression [12, 23, 60]. Each five dimensions (items) are rated on five levels (1 = no problems, slight problems, moderate problems, severe problems, and 5 = extreme problems/unable to) [61]. EQ-5D-5L utilities were computed indirectly using the Hungarian value set, based on preferences of the general population [25]. Worse than dead values (n = 15) were truncated to 0 for harmonization of the utility range. EQ-VAS was part of the questionnaires, but not included into the current study.

*(3–4)* The *DLQI* is the first developed and most commonly used dermatology-specific HRQoL measure that has more than 90 languages versions and validated in 30 + skin diseases (e.g., acne, melasma, morphea, urticaria) [4, 62–64]. DLQI has 10 items, which cover six health domains (symptoms and feelings, daily activities, leisure, work and school, personal relationships and treatment) [65]. Each item is rated on 0–3 severity levels (L1-3), the higher DLQI score (max 30 – min 0 points) indicates a worse HRQoL impact [66]. Patient’s responses were converted to utilities by applying two different approaches:

(3) *Mapping DLQI item responses to utilities* (mDLQI) with a regression-based algorithm, using the United Kingdom’s (UK) value set according to the following equations [30]:

$$EQ - 5D - 3L\left( x \right) = EQ5D3L\left( {1 = full \ health} \right) \ if \ DLQI = 0$$

$$EQ - 5D - 3L\left( x \right) = mDLQI\left( x \right) = \beta_{0} + \beta_{1} DLQI score + \beta_{2} sex + \beta_{3} age \ if \ DLQI \ne 0$$

(4) A second approach used *value set based generation of utilities* (vDLQI) from DLQI responses, that relied on the evaluations of the Hungarian general public [20, 21]. The estimation uses a censored regression, adding cumulative disutilities according to the DLQI item’s level score (L1,2,3) to the intercept [31]:

$$vDLQI(x)=0.873- {\sum }_{i=10}^{i}{\sum }_{l=3}^{l}disutility$$

where ‘i’ denotes the 10 items of the questionnaire and ‘l’ represents the item’s level score.

### Clinical outcome measures

In addition to sociodemographic characteristics (age, sex, educational level, employment status), all surveys contained additional health-related information (disease duration, outpatient care visits in the past 3 months and disease severity). In each survey, disease severity was assessed by physicians using validated tools in the respective condition: Scoring Atopic Dermatitis (SCORAD) in atopic dermatitis [56], the modified Sartorius Score (mSS) in hidradenitis suppurativa [8], the Autoimmune Bullous Skin Disorder Intensity Score (ABSIS) in pemphigus [50] and the Psoriasis Area Severity Index (PASI) in psoriasis [48]. Disease-specific severity indices (ABSIS, PASI, mSS, SCORAD) were normalized to a range of 0–1 scores (with 1 referring to the worst possible severity) named the “skin disease severity” score to make disease severity ratings comparable.

### Statistical analysis

Descriptive statistics were computed for the pooled sample and according to diagnoses. Mean, standard deviation (SD), median, interquartile range (IQR), ceiling and floor effects were calculated for TTO, EQ-5D, mDLQI and vDLQI utilities. Mean utility scores across groups were compared by Kruskal‒Wallis or Mann‒Whitney nonparametric tests. Pearson’s (r) correlation of utilities was calculated to examine their association.

### Agreement measure

Bland‒Altman plots and intraclass correlation coefficients (ICCs) were used to assess measurement agreement across the four utility estimates [67]. The Bland‒Altman method investigates the relationship of discrepancies between two measurements to detect proportional bias, that is, a systematic difference between two instruments, meaning that one measurement gives consistently higher/lower values. The Bland‒Altman plots are often applied to visualize limits of agreement (LOA: *mean difference* ± 1.96*SD) between different instruments that measure the same construct (in our study: health utility) [68].

To compute ICC values, a two-way random model (randomizing both subject and instrument effects, with absolute agreement) was applied. ICC reflects the reliability of measurements, representing the proportion of total variance due to the variations between cluster members, and it indicates poor, moderate and strong agreement if ICC ≤ 0.50, 0.51–0.74 and 0.75–1, respectively [69].

### Regression analysis of the impact of sociodemographic factors on utilities

The association of sociodemographic and disease-related variables on TTO, EQ-5D, mDLQI and vDLQI utility estimates was analysed by multivariate Tobit regression model to treat probable (left-sided) skewness in utility data distribution while still assuming a linear relationship between the dependent variable (health utility) and independent variables [70–72]. Respondent’s choice of the highest possible score is often reported in literature with TTO and EQ-5D utility measurements, leading to the recommendation to censor the utility score at 1.0 (or at the theoretical maximum in our case) [73, 74]. Heteroscedastic utility distribution was controlled with robust standard errors. Three continuous and five dummy coded explanatory variables were included in the models as independent variables: age (in years), sex (male reference coded), level of education (primary reference coded), employment status (unemployed reference coded), disease duration (in years), outpatient care visits (yes or no), skin disease severity (normalized score), and type of skin disease (pemphigus reference coded). The regression equation is the following in all four models:

$$\left( {health \, state} \right) \, utility_{i} = \beta_{0} + \beta_{i} Age + \beta_{i} Sex + \beta_{i} Educ + \beta_{i} Empl + \beta Disdur \, + \beta_{i} Outpat \, + \beta_{i} Skincond + \gamma_{i} Diseasetype + \epsilon_{i} = utility_{i} * \, if \, utility_{i} * < \ maximum\, otherwise, \, utility_{i} = 0 \, if \, utility_{i} * \ge \ maximum$$

## Results

### Population characteristics

Overall, the responses of 765 patients with chronic dermatological conditions were analysed (atopic dermatitis = 218; hidradenitis suppurativa = 200; pemphigus = 109; psoriasis = 238). The proportion of females was 47.3%, and the age ranged between 18 and 93 years, with a mean of 41.5 (SD = 16.2). Atopic dermatitis patient sample was the youngest (31.3), while pemphigus patients were on average 57.2 years old. The average disease duration was 12.8 (SD = 12.6) years, the highest being in atopic dermatitis (19.0) and the lowest in pemphigus (3.8). Altogether, 435 (56.9%) patients had used outpatient care services in the past 12 months. Skin disease severity scores in atopic dermatitis, hidradenitis suppurativa, psoriasis and pemphigus were 0.51, 0.22, 0.18 and 0.14, respectively. The mean total DLQI score was 9.9 (SD = 8.4), the highest being in atopic dermatitis (13.4) and the lowest in pemphigus (5.4) (Table 2).

**Table 2:** Population characteristics

Variables	n (%) or mean (SD)				
	Total	Pemphigus	Psoriasis	Hidradenitis suppurativa	Atopic dermatitis
Characteristics					
Total	765 (100)	109 (14.2)	238 (31.1)	200 (26.1)	218 (28.5)
Age in years	41.5 (16.2)	57.1 (14.8)	47.4 (15.2)	37.1 (12.4)	31.3 (11.7)
Sex					
Female	362 (47.3)	70 (64.2)	89 (37.4)	77 (38.5)	126 (57.8)
Male	403 (52.7)	39 (35.8)	149 (62.6)	123 (61.5)	92 (42.2)
Education (missing n = 3)					
Primary	93 (12.2)	22 (20.2)	19 (8.0)	40 (20.0)	12 (5.5)
Secondary	431 (56.3)	58 (53.2)	132 (55.5)	129 (64.5)	112 (51.4)
Tertiary	238 (31.1)	29 (26.6)	87 (36.6)	30 (15.0)	92 (42.2)
Employment status					
Full-time employed	383 (50.1)	41 (37.6)	121 (50.8)	117 (58.5)	104 (47.7)
Part-time employed	37 (4.8)	4 (3.7)	13 (5.5)	9 (4.5)	11 (5.0)
Student	83 (10.8)	1 (0.9)	7 (2.9)	21 (10.5)	54 (24.8)
Retired	97 (12.7)	38 (34.9)	44 (18.5)	4 (2.0)	11 (5.0)
Disability pensioner	63 (8.2)	15 (13.8)	26 (10.9)	14 (7.0)	8 (3.7)
Unemployed	53 (6.9)	6 (5.5)	13 (5.5)	25 (12.5)	9 (4.1)
Other (e.g. housewife)	49 (6.4)	4 (3.7)	14 (5.9)	10 (5.0)	21 (9.6)
Outpatient care use (missing n = 6)					
Yes	435 (56.9)	54 (49.5)	211 (88.7)	71 (35.5)	99 (45.4)
No	324 (42.7)	55 (50.5)	27 (11.3)	126 (63.0)	116 (53.2)
Disease duration in years (missing n = 3)	12.8 (12.6)	3.8 (4.9)	18.1 (12.3)	4.8 (6.7)	19.0 (12.9)
Skin disease severity*	0.28 (0.24)	0.14 (0.20)	0.18 (0.19)	0.22 (0.18)	0.51 (0.21)
DLQI** score (0–30) (missing n = 4)	9.9 (8.4)	5.4 (6.8)	7.1 (7.4)	(8.1)	13.4 (8.5)

*Disease severity score was given as normalization of ABSIS, PASI, mSS and SCORAD disease-severity measures (scores ranging between 0–1, where the higher value refers to worse state)

**DLQI Dermatology Life Quality Index (measurement tool)

### Utility descriptive statistics and data distribution

**Fig. 1:**
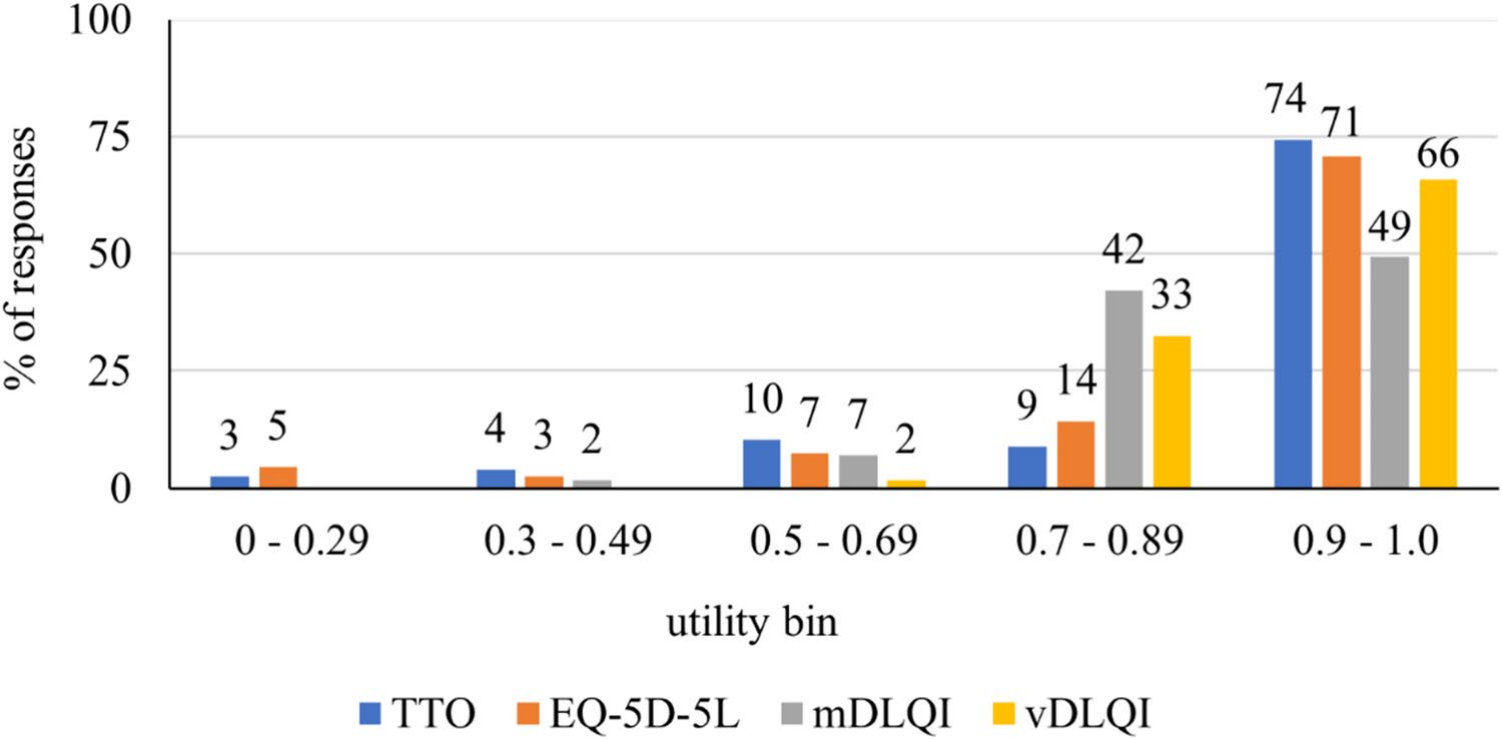
Distribution of all patient responses according to the four health state utility measurements. *TTO time trade-off, *mDLQI* mapping-based dermatology life quality index utility, *vDLQI* value set-based dermatology life quality index utility

The mean (SD) TTO, EQ-5D-5L, mDLQI and vDLQI utilities were 0.83 (0.24), 0.81 (0.24), 0.77 (0.14) and 0.81 (0.08), respectively. TTO values showed the highest median (IQR) utility score of 0.95 (0.74–1), followed by EQ-5D-5L with 0.89 (0.75–0.97), vDLQI with 0.84 (0.76–0.87) and mDLQI with 0.79 (0.70–0.86). Utility data were skewed to the left by all utility assessment methods, and TTO and EQ-5D-5L peaked at 1. The ceiling effect was the highest in vDLQI (n = 503, 65.8%), and TTO produced the highest proportion of ‘maximum’ utilities (n = 331, 45.3%). However, this proportion was much lower (n = 187, 24.6%) for the EQ-5D-5L and mapping converted mDLQI utility (n = 101, 13.3%). The vDLQI utility had a range of 0.57 to 0.87 and the mDLQI utility was the closest to a normal distribution minimum–maximum scores ranged between 0.25 and 0.98 (Fig. 1).

Overall, 4 × 18 utility scores of the 4 measures by 5 category variables (disease type, sex, education level, employment status, outpatient care use) were computed, whereby TTO produced the highest mean utility in 10 (56%) cases. In the remaining five (28%) and 3 (17%) cases, the vDLQI and the EQ-5D-5L resulted in the highest mean utility, respectively.

Psoriasis patients had the highest and pemphigus the lowest mean utility according to TTO (0.91 vs 0.72). Similarly, the highest mean EQ-5D-5L utility was observed in psoriasis, but the lowest was observed in hidradenitis suppurativa. Both DLQI utility estimations produced the same severity order: the lowest mean utility was found in atopic dermatitis and the highest in pemphigus and psoriasis. Among the four diseases, the highest range of mean utilities was in TTO (0.19), which was fairly lower in EQ-5D-5L (0.08), mDLQI (0.06) and vDLQI (0.05). Women had higher mean utility compared to men only according to TTO (0.84 vs. 0.83), while men had higher mean utility with the EQ-5D-5L (0.82 vs 0.81), mDLQI (0.79 vs 0.75) and vDLQI (0.82 vs 0.79).

Nonparametric tests revealed statistically significant differences in mean scores according to disease type, educational level and employment status (p < 0.05). Patients who were higher educated and full- or part-time employed had significantly higher mean utility scores according to all measures. Male sex was significantly associated with a higher mDLQI and vDLQI utility. (Table 3).

**Table 3:** Comparison of mean utility scores among patient subgroups

Variable*	TTO utility				EQ-5D-5L utility				mDLQI utility				vDLQI utility			
	n	Mean (SD)	Median	p value	n	Mean (SD)	Median	p value	n	Mean (SD)	Median	p value	n	Mean	Median	p value
Total	730	0.83 (0.24)	0.95	–	761	0.81 (0.24)	0.89	–	761	0.77 (0.13)	0.79	–	764	0.81 (0.08)	0.84	–
Disease																
psoriasis	238	0.91 (0.16)	1	< *0.001*	237	0.84 (0.22)	0.92	< *0.001*	238	0.80 (0.11)	0.81	< *0.001*	238	0.83 (0.07)	0.87	< *0.001*
atopic dermatitis	193	0.85 (0.22)	0.95		218	0.83 (0.21)	0.89		218	0.74 (0.14)	0.76		218	0.78 (0.09)	0.81	
hidradenitis suppurativa	194	0.78 (0.27)	0.90		198	0.76 (0.28)	0.86		198	0.75 (0.13)	0.78		200	0.79 (0.09)	0.82	
pemphigus	105	0.72 (0.32)	0.90		108	0.82 (0.25)	0.91		107	0.80 (0.10)	0.83		108	0.84 (0.06)	0.87	
Sex																
female	347	0.84 (0.24)	0.95	0.490	360	0.81 (0.23)	0.89	0.112	360	0.75 (0.13)	0.77	< *0.001*	361	0.79 (0.09)	0.83	< *0.001*
male	383	0.83 (0.24)	0.95		401	0.82 (0.25)	0.92		401	0.79 (0.12)	0.81		403	0.82 (0.07)	0.85	
Education																
primary	89	0.75 (0.30)	0.90	< *0.001*	91	0.69 (0.31)	0.79	< *0.001*	91	0.74 (0.12)	0.75	*0.003*	92	0.78 (0.09)	0.80	< *0.001*
secondary	409	0.81 (0.25)	0.95		430	0.81 (0.24)	0.89		430	0.77 (0.12)	0.78		431	0.80 (0.08)	0.84	
tertiary	230	0.90 (0.18)	1		237	0.86 (0.18)	0.92		237	0.78 (0.12)	0.81		238	0.82 (0.08)	0.85	
Employment status																
full-time employed	366	0.85 (0.23)	0.95	*0.025*	382	0.86 (0.20)	0.92	< *0.001*	381	0.79 (0.12)	0.81	< *0.001*	383	0.81 (0.08)	0.85	< *0.001*
part-time employed	35	0.89 (0.12)	0.95		37	0.81 (0.24)	0.85		37	0.77 (0.13)	0.79		37	0.80 (0.09)	0.84	
student (university)	82	0.80 (0.25)	0.90		83	0.84 (0.21)	0.91		83	0.78 (0.13)	0.79		83	0.79 (0.08)	0.82	
retired	94	0.83 (0.27)	1		96	0.77 (0.25)	0.88		96	0.74 (0.09)	0.74		96	0.83 (0.06)	0.85	
disability pensioner	60	0.76 (0.30)	0.90		62	0.68 (0.29)	0.76		63	0.72 (0.15)	0.75		63	0.78 (0.10)	0.81	
unemployed	49	0.78 (0.25)	0.90		52	0.69 (0.32)	0.83		52	0.74 (0.12)	0.78		53	0.78 (0.09)	0.81	
other	44	0.90 (0.19)	1		49	0.79 (0.26)	0.88		49	0.73 (0.15)	0.77		49	0.78 (0.10)	0.84	
Outpatient care use																
yes	416	0.84 (0.25)	0.95	0.130	433	0.81 (0.25)	0.88	0.805	433	0.77 (0.13)	0.79	0.872	435	0.81 (0.08)	0.85	0.137
no	309	0.83 (0.23)	0.95		322	0.83 (0.22)	0.91		322	0.77 (0.11)	0.79		323	0.81 (0.08)	0.84	

*Significant (p < 0.05) difference in mean utilities among subgroups are highlighted with italic p values

*TTO* time trade-off, *mDLQI* mapping-based dermatology life quality index, *vDLQI* value set-based dermatology life quality index

Weak to strong significant correlations were observed between the utility scores. The strongest correlation was found between the two DLQI utility estimates (r = 0.900; p < 0.01). The EQ-5D-5L was moderately correlated with mDLQI and vDLQI utility (r = 0.598 and 0.556; p < 0.01) and weakly correlated with TTO (r = 0.287; p < 0.01). TTO showed the weakest correlation with the mDLQI and vDLQI (r = 0.244 and 0.257, p < 0.01). (Table 4).

**Table 4:** Pearson’s correlation coefficients and ceiling/floor effect of utility measures

Measure*	*TTO*	*EQ-5D-5L*	*mDLQI*	*vDLQI*
*TTO*	*1*			
*EQ-5D-5L*	*0.287*	*1*		
*mDLQI*	*0.244*	*0.598*	1	
*vDLQI*	*0.257*	*0.556*	*0.900*	1
Ceiling effect (%)	45.3%	24.6%	13.3%	65.8%
Floor effect (%)	1.8%	3.3%	0.1%	1.7%
Variance	0.06	0.06	0.16	0.01

*All correlations were significant (at p < 0.01 level). Ceiling and floor effect show the proportion of responses on best and worst endpoints (1 & 0) of the utility scale. Variance shows the dispersion of utility score among individuals, the higher it is the more heterogeneous the patient’s utility is

### Measurement agreements

**Fig. 2:**
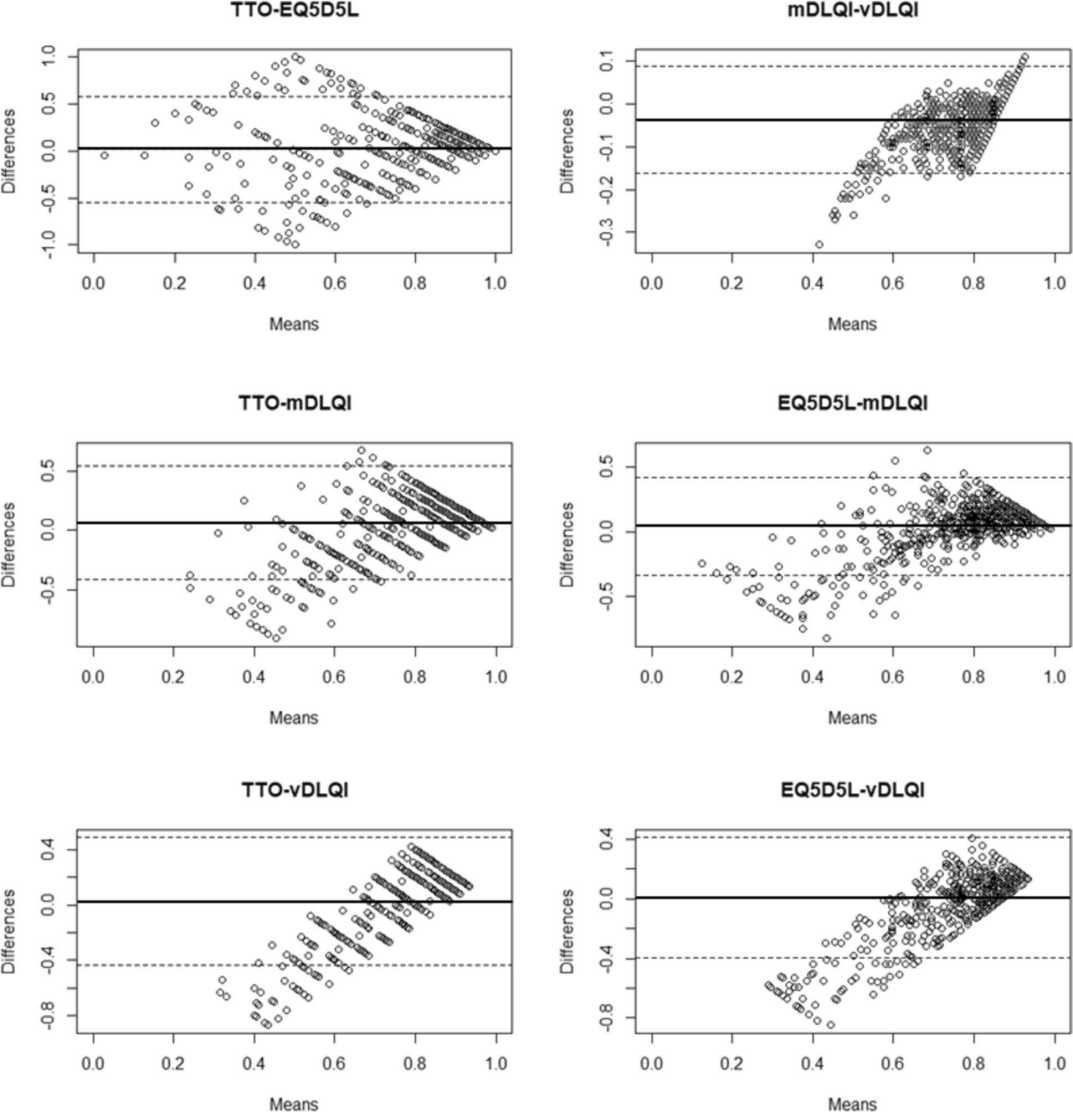
Bland–Altman plots showing the agreement between the four health state utility measurements. interpretation: The horizontal solid and dashed lines indicate the mean differences and 95% confidence intervals (LOA), showing the absolute agreement between each pair of utility measurements. In case of TTO-EQ5D5L the funnel shaped scatter indicates measurement agreement, despite the non-consistent variability TTO agrees with EQ-5D-5L in capturing utility values. While the most obvious case of disagreement is between TTO and mDLQI, where the wide cluster of patient responses scattering outside the LOA at worse health states suggests that mDLQI might under/overestimates health utilities (that pattern is visible across all DLQI-based utility measures). Plots imply that TTO and EQ-5D-5L were reliable in measuring dermatological patients’ health utility, while DLQI-based measures failed in capturing worse/mild health states utility accurately. *TTO time trade-off, *mDLQI* mapping-based dermatology life quality index utility; *vDLQI* value set-based dermatology life quality index utility

The Bland‒Altman analysis revealed measurement agreement only between TTO and EQ-5D-5L (mean difference: 0.016; SD = 0.287; p = 0.124), with moderate test–retest reliability between individuals (ICC = 0.445; 95% CI: 0.36–0.52; p < 0.001). Agreement was established between the EQ-5D-5L and vDLQI (mean difference: 0.008; SD = 0.206; p = 0.317), although the regression revealed a high level of proportional bias. TTO and mDLQI/vDLQI as well as EQ-5D and mDLQI/vDLQI measures showed the presence of systemic proportional bias. Although absolute agreement between individuals in mDLQI and vDLQI measures was strong (ICC = 0.872; p < 0.001), moderate in EQ-5D-5L and mDLQI/vDLQI measures (ICC = 0.646 and 0.505; p < 0.001), but rather poor between TTO and the two DLQI utility measures (ICC = 0.314 and 0.263; p < 0.001). Bland‒Altman plots further supported the extent of disagreements between the TTO and mDLQI/vDLQI measures. Similar to the EQ-5D-5L and DLQI utility measures, the scores misfit limits of agreement, especially at the lower end of the utility scale, indicating presence of systemic disagreement is worse health states. (Table 5 and Fig. 2).

**Table 5:** Measurement agreement results

measures	∆ TTO-EQ5D5L utility	∆ TTO-mDLQI utility	∆ TTO-vDLQI utility	∆ EQ5D5L -mDLQI utility	∆ EQ5D5L -vDLQI utility	∆ mDLQI-vDLQI utility
Bland‒Altman plot results						
Mean difference (t test p value)	0.016 (0.124)	0.062 *(*< *0.001*)	0.025 (*0.004*)	0.045 (< *0.001*)	0.008 (0.317)	0.037 (< *0.001*)
Standard deviation of differences	0.287	0.243	0.235	0.193	0.206	0.063
Limits of agreement (95% lower–upper CI)	− 0.546 to 0.579	− 0.415 to 0.538	− 0.436 to 0.486	− 0.333 to 0.423	− 0.396 to 0.411	−0.086 to 0.161
Regression β coef. (p value)	0.033 (0.546)	0.999 (< 0.001)	1.397 (< 0.001)	0.770 (< 0.001)	1.187 (< 0.001)	−0.446 (< 0.001)
ICC results						
ICC*	0.445	0.314	0.263	0.646	0.505	0.872
95% lower–upper CI	0.358–0.520	0.205–0.408	0.148–0.362	0.582–0.700	0.430–0.571	0.728–0.927
p value	< 0.001	< 0.001	< 0.001	p < 0.001	p < 0.001	p < 0.001

*Intraclass correlation coefficient (ICC): two-way random model with absolute agreement among measures was used. If mean difference t test is significant, the measurements utility scores significantly differ, showing no agreement

### Association between sociodemographic factors, clinical characteristics and utility estimates

Out of the four tobit models, the greatest proportion of variance was explained in TTO utility by the observed sociodemographic and clinical characteristics variation (R^2^ = 38.0%; σ = 0.12). The TTO utilities were significantly higher among higher educated patients (β = 0.15) and disease severity was negatively associated (β = −0.45) with TTO utilities. All HSU estimates had significant relationship with the skin disease severity, however only the TTO method differentiated utility values according to disease type (atopic dermatitis: β = 0.35, psoriasis: β = 0.29, hidradenitis suppurativa: β = 0.13). EQ-5D-5L utility elevated in patients with secondary and higher education background (β = 0.08 and 0.13) and in those being full-time employed (β = 0.09), decreased among hidradenitis suppurativa patients (β = −0.12) or in those having more severe skin disease (β = −0.49). Older age, female sex, and living in hidradenitis suppurativa slightly but significantly decreased mDLQI utilities. Slightly higher vDLQI utilities were observed in patients with tertiary education (β = 0.03), and lower in female, hidradenitis suppurativa patients and those having worse disease severity. (Table 6).

**Table 6:** Effect of patients’ sociodemographic and clinical characteristics on utility estimates: tobit regression results

Variables****	TTO utility		EQ-5D-5L utility		mDLQI utility		vDLQI utility	
	β coefficient (SE)	p value	β coefficient (SE)	p value	β coefficient (SE)	p value	β coefficient (SE)	p value
Age	0.002 (0.00)	0.138	− 0.001 (0.00)	0.310	**− 0.002 (0.00)**	** < 0.001**	0.001 (0.00)	0.054
Sex	0.022 (0.03)	0.451	− 0.038 (0.02)	0.063	**− 0.050 (0.01)**	** < 0.001**	**− 0.035 (0.01)**	** < 0.001**
Secondary education	0.036 (0.05)	0.450	**0.079 (0.03)**	**0.038**	0.009 (0.01)	0.480	0.015 (0.01)	0.233
Higher education	**0.152 (0.05)**	**0.004**	**0.129 (0.04)**	**0.002**	0.021 (0.01)	0.135	**0.034 (0.01)**	**0.014**
Full-time employed	0.036 (0.05)	0.463	**0.094 (0.05)**	**0.038**	0.012 (0.02)	0.429	0.015 (0.02)	0.292
Part-time employed	0.053 (0.06)	0.389	0.040 (0.07)	0.492	− 0.007 (0.02)	0.761	0.003 (0.02)	0.898
Retired	− 0.032 (0.08)	0.672	− 0.041 (0.05)	0.533	− 0.013 (0.02)	0.508	− 0.011 (0.02)	0.615
Disability pensioner	− 0.080 (0.07)	0.275	− 0.064 (0.06)	0.301	− 0.029 (0.02)	0.174	− 0.029 (0.02)	0.146
Student	− 0.014 (0.07)	0.830	0.091 (0.05)	0.072	0.015 (0.02)	0.391	0.024 (0.02)	0.180
Other employment	0.126 (0.08)	0.098	0.033 (0.06)	0.583	− 0.007 (0.02)	0.774	− 0.001 (0.02)	0.974
Disease duration (years)	0.000 (0.00)	0.890	− 0.001 (0.00)	0.423	0.000 (0.00)	0.662	0.000 (0.00)	0.649
Outpatient care use (y/n)	− 0.020 (0.03)	0.518	− 0.025 (0.02)	0.271	− 0.013 (0.01)	0.141	− 0.008 (0.01)	0.366
Skin disease severity (normalized)	**− 0.447 (0.08)**	** < 0.001**	**− 0.486 (0.06)**	** < 0.001**	**− 0.293 (0.03)**	** < 0.001**	**− 0.256 (0.02)**	** < 0.001**
Psoriasis	**0.293 (0.06)**	** < 0.001**	0.017 (0.04)	0.684	− 0.015 (0.01)	0.248	− 0.001(0.02)	0.959
Hidradenitis suppurativa	**0.130 (0.06)**	**0.022**	**− 0.119 (0.04)**	**0.002**	**− 0.070** (0.01)	** < 0.001**	**− 0.057 (0.02)**	** < 0.001**
Atopic dermatitis	**0.352 (0.07)**	** < 0.001**	0.073 (0.05)	0.132	− 0.005 (0.02)	0.798	0.006 (0.02)	0.746
Constant	0.682 (0.10)	< 0.001	0.952 (0.07)	< 0.001	0.961 (0.03)	< 0.001	0.886 (0.03)	< 0.001
Regression model indices								
Squared multiple correlation (R^2^)	0.380		0.317		0.300		0.338	
Observations (n)***	724		753		753		756	
Uncensored	394		569		752		459	
Right censored at max	330		184		1		297	
Log likelihood	− 362.9		− 176.6		679.7		235.9	
Prob > (chi)	< 0.001		< 0.001		< 0.001		< 0.001	
Variance (sigma)	0.120		0.066		0.010		0.009	

*Male group, primary education, unemployment, pemphigus disease was set as reference category (to 0) when coding dummies

**mDLQI refers to mapping-based DLQI utility, vDLQI refers to value set-based DLQI utility

***TTO (time trade-off measurement method) has fewer observations because the inconsistent responses were removed, which is described in detail in previous studies

****Coefficients and corresponding p-values in bold indicate independent variables that have a significant effect on health state utility

## Discussion

This is the first study comparing different direct and indirect (TTO and EQ-5D-5L, mDLQI, vDLQI) methods for generating health states utilities in patients with atopic dermatitis, hidradenitis suppurativa, pemphigus, psoriasis. Our analysis revealed measurement agreement only between TTO and EQ-5D-5L utilities. Mean differences between TTO and EQ-5D-5L (0.016) showed minimal bias, although the large SD of differences (0.29) indicate non-consistent variation in the agreement. The moderate ICC (0.45) also warns to the volatility of measurement agreement between TTO and EQ-5D-5L. No agreement was found between the TTO-mDLQI/vDLQI and EQ-5D-5L ‒ mDLQI/vDLQI, showing considerable differences across measures especially in the more severe health states. EQ-5D-5L and mDLQI/vDLQI utility measures correlated strongly (r = 0.598 and 0.556; p < 0.01), while TTO and EQ-5D-5L/mDLQI/vDLQI weakly (r = 0.287; 0.244; 0.257; p < 0.001), which further affirms contrast between direct vs. indirect utility measurements. The observed agreement between EQ-5D-5L and vDLQI may be attributable to the similarity of national value sets, representing the preferences of the Hungarian population. The TTO showed superiority in differentiating the utilities across diseases, while the regression analysis revealed similar impact of decreasing utilities with worse skin condition severity (ABSIS, PASI, mSS, SCORAD) and socioeconomic variables (age, sex, level of education and employment status) effect on HSU as in previous studies [75–77].

In subgroups TTO was the highest in ten out of eighteen cases, contrary to mDLQI utility being the lowest in fifteen subgroups. Supposedly, out of the four investigated skin diseases, the pemphigus is the most and atopic dermatitis is the least severe overall health state, as resulted by the direct (TTO) evaluation of patient’s current health. Conflictingly, atopic dermatitis had the lowest and pemphigus the highest DLQI utility values (mDLQI: 0.74 vs 0.80 and vDLQI: 0.78 vs 0.84). Such pattern might be associated with the principal difference between direct and indirect measurement methods (a demanding overall health assessment required by the TTO tasks vs. rapid rating of items covering narrower health domains by DLQI).

While the TTO task requests patients’ assessment (by demanding to choose between quality vs quantity of life) of their current overall health, until the DLQI instrument’s 10 items focus solely on skin problems (e.g.: itchiness, dress wearing, embarrassment) thus capturing skin-condition related domains of health. Considerable differences between the TTO and DLQI elicited utilities may be associated with the patient sample’s mean age, where pemphigus patients had a mean age of 57.1, with a mild skin-condition severity reflected by a DLQI total score of 5.4 and skin disease severity normalized score (0.14), although had lowest TTO mean utility (0.72) among the investigated diseases. In contrast, atopic dermatitis patients had a mean age of 31.3, and a mean TTO utility of 0.85, parallelly with the worst DLQI total (13.4) and skin disease severity (0.51) score. There is evidence of worse HRQoL impact of skin diseases on younger adult dermatology patients, potentially due stronger impact of skin symptoms on social and partnership related health domains (DLQI items 5, 8, 9) as compared to older aged patients [78, 79]. While generally lower TTO utility is associated with ageing, when an overall assessment of health is likely to capture heterogeneous health states including skin symptoms and comorbidities (and the incremental value of quantity of life is higher) [6, 80].

Both direct and indirect utility measurement have shortcomings. Complicated tasks such as the TTO encourage the use of heuristics, relativization that mismatch with blur and mutable preferences [81]. Pattern answering, carelessness of respondents or design issues with the instrument items/rating scale may cause shortcomings for indirect utility valuations [82]. Using dissimilar population values set to weight HRQoL items or converting HRQoL instrument item scores into utility may also present pitfalls [33, 83, 84]. Converted utilities in dermatology face a second shortfall; that is, except for the DLQI, there is currently no other skin disease-specific HRQoL instrument offering a mapping algorithm or value set [85]. While there are multiple cancer-specific instruments available for indirect utility elicitation [86]. In addition, more studies are concerned that scoring the DLQI items as ‘0’, both equivalent to “not relevant” or “not at all”, may bias the disease severity by underestimating the DLQI score [54, 87, 88].

As opposed to the utility study in dermatology of Liu et al. [9] and in line with the more-publicized evidence in other chronic diseases [44, 89, 90], our findings revealed higher mean utility scores elicited with the direct measurement method. The TTO resulted in the highest mean HSU scores in most cases and generally exceeded the m/vDLQI-based utility means. Health evaluations might suggest using societal preference based on indirect utilities elicited with national value sets, but for clinicians, the directly measured utilities of patients may enfold more complete information about the HRQoL in a given skin disease.

### Limitations

The study has not considered (1) that the algorithm converted mDLQI utility was optimized for the UK EQ-5D-3L values though doesn’t represent the preferences of the Hungarian population. Another issue is (2) the dissimilar nature of diseases, which may differently affect HRQoL domains. The TTO utilities (3) reflect patient’s current health and not the general public’s vignette-based (hypothetical) valuations. Converting the DLQI item scores into utilities in four fairly different conditions limits the comparability of HSU measurement results (e.g., the DLQI score in pemphigus was 5.4; thus, the converted vDLQI utility yielded 0.83, while the initial atopic dermatitis DLQI score was 13.4, which had an mDLQI utility of 0.74). In comparison, the TTO utility was the lowest in pemphigus (0.72), while it was significantly higher in atopic dermatitis (0.85). This phenomenon highlights that DLQI items describe a certain set of skin condition-related problems (e.g., itching, appearance, social discernment), while TTO focuses on respondents’ overall health state associated with a disease, but there is not necessarily a match between the two.

### Conclusions

This is the first study that investigated measurement agreement between direct and indirect generic/indirect dermatology-specific utility measurements. Measurement agreement was found only between TTO and EQ-5D-5L and both showed disagreement with the DLQI utility estimates. The abyss found between the TTO method and DLQI converted utility in atopic dermatitis and pemphigus warns to compare DLQI-based utilities in different dermatological conditions with extreme caution. Clinical practice should consider the application of direct utility measurements to assess patient’s overall HRQoL.

## Data Availability

The datasets used and analysed during the study are available from the corresponding author on reasonable request.
